# Complications After Intramedullary Fixation Treatment of Patients With Osteogenesis Imperfecta: Telescopic Versus Non-Telescopic Implants

**DOI:** 10.7759/cureus.45376

**Published:** 2023-09-16

**Authors:** Tayfun Bacaksiz, Ihsan Akan

**Affiliations:** 1 Department of Orthopedics and Traumatology, Izmir Katip Celebi University Ataturk Training and Research Hospital, Izmir, TUR

**Keywords:** complication, non-telescopic implants, telescopic nail, intramedullary nail, osteogenesis imperfecta

## Abstract

Introduction

The treatment of musculoskeletal pathologies in osteogenesis imperfecta aims to provide the maximum possible function by preventing bone fractures and progressive deformities. Despite the development of implants used in the surgical treatment of osteogenesis imperfecta, there is no consensus on the most appropriate method for the correction of skeletal deformities. The aim of this study was to compare telescopic and non-telescopic implants in terms of postoperative complications.

Methods

Twenty-three patients who were operated on for the diagnosis of osteogenesis imperfecta between 2005 and 2018 were retrospectively analyzed. Demographic data, follow-up times, and the total number of surgeries and complications were recorded. The operated bones were divided into two groups according to whether the intramedullary fixation material used was telescopic or not.

Results

Twenty-one of 23 patients were included in the study due to the use of intramedullary fixation material in the operation. The mean age was 10.1 ± 2.9 years, and the mean follow-up period was 8.9 ± 3.5 years. Intramedullary fixation was applied to 43 long bones in 21 patients due to fracture or deformity. At least one complication was encountered in nine of 14 bones with telescopic implants and in 27 of 29 bones with non-telescopic implants. Major complications requiring surgical treatment were seen in seven bones of the telescopic implant group and 27 bones of the non-telescopic implant group.

Conclusion

The use of telescopic implants relatively reduces the complication rate and the need for repetitive surgery in patients with a diagnosis of osteogenesis imperfecta. However, the number of complications is still as high as with non-telescopic implants.

## Introduction

Osteogenesis imperfecta (OI) is a congenital connective tissue disease, and the main pathology is a defect in type 1 collagen synthesis [1]. Therefore, many systems in the body are affected, and a multidisciplinary approach is required. In these patients, aortic and mitral valve insufficiency, malignant hyperthermia, hearing problems, impaired dental health, and vascular problems can be seen. However, musculoskeletal findings are the most severe. Among these, recurrent microfractures and secondary deformities have special importance because they both affect the mobility of the patients and are a source of chronic pain [2].

The treatment of musculoskeletal pathologies aims to provide the maximum possible function by preventing bone fractures and progressive deformities. In mild forms, conservative treatment of fractures and deformities may be sufficient, while surgery is the only option for rehabilitation in severe forms. The use of intramedullary fixation is the most preferred method to achieve this. Miller was one of those who used rods as non-telescopic intramedullary fixation after multiple osteotomies for deformity correction [3]. The most important disadvantage of non-telescopic implants is that they reduce the problem of fracture or deformity until the bone heals. With the lengthening of the bone, the implant cannot accompany it, and complications occur, especially in the areas close to the implant tips. Over time, Bailey and Dubow telescopic rods began to be used to accommodate the lengthening bone [4,5]. This was followed by the Fassier-Duval telescopic implant [6]. The advantage of telescopic implants is that they have elongation properties in parallel with bone growth and thus prevent secondary deformities for a much longer time [7,8]. However, this technique is very laborious, and in addition, many complications have also been reported [9-11].

Although the use of telescopic implants is increasing day by day, there is no consensus on the most appropriate method for the correction of skeletal deformities in OI. The aim of this study is to present our experience with telescopic and non-telescopic intramedullary fixation methods used in patients with osteogenesis imperfecta, to compare the complications that can be seen, and to highlight the points to be considered in the development of telescopic implants.

## Materials and methods

Patients and methods

Twenty-three patients who were operated on for the diagnosis of osteogenesis imperfecta between 2005 and 2018 were retrospectively analyzed. All patients were operated on in the same center by the same team, under the leadership of two different pediatric orthopedic specialists. Inclusion criteria for the study were defined as having a diagnosis of osteogenesis imperfecta and using only intramedullary fixation material in the surgery. One of the pediatric orthopedic specialists preferred fixation with traditional methods and used non-telescopic intramedullary material for the patients who decided to have an operation with the same indication, while the other used a telescopic implant for the same patient group. Insufficient follow-up period, use of any fixation method other than an intramedullary implant, and use of medical drugs such as hormone therapeutics and bisphosphonates that would disrupt the standardization of the study were determined as exclusion criteria.

All patients were followed up with a splint for six weeks after surgery. After splint removal, the same physical therapy programs were applied to all patients. They were allowed to walk with a partial load on the sixth weekend and a full load on the third month. Radiographs were seen at regular intervals for union, recurrence of deformity, and complication follow-up. Patient information was obtained retrospectively from patient files and radiological databases. Demographic data, follow-up times, and the total number of surgeries and complications were recorded. The operated bones were divided into two groups according to whether the intramedullary fixation material used was telescopic or not. Complications for both groups were documented as those requiring surgical treatment (a major complication) and those not requiring surgical treatment (a minor complication).

The study was approved by the Institutional Review Board Committee of the Faculty of Medicine, İzmir Katip Çelebi University (Decision number: tel:0523-2022), Turkey, and has been performed according to the ethical standards of Helsinki.

Statistical analysis

The statistical analysis was performed using Statistical Package for the Social Sciences version 21.0 (SPSS Inc., Chicago, IL, USA). Data were given as mean ± standard deviation (minimum-maximum) for continuous variables. Categorical variables were compared with Chi-square or Fisher exact test testing, as appropriate. The Mann-Whitney U Test has been used for numerical data in group comparisons for the reason that the group sample size was less than 30 and the data were not normally distributed. The results were evaluated at the 95% confidence interval and the accepted significance level of p < 0.05.

## Results

Twenty-one of 23 patients were included in the study due to the use of intramedullary fixation material in the operation. Two of them were excluded from the study on account of osteosynthesis with a plate after a fracture. Of 21 patients, 12 (57%) were males and nine (43%) were females. The mean age was 10.1 ± 2.9 years (range: five to 14 years), and the mean follow-up period was 8.9 ± 3.5 years (range: three to 15 years).

Intramedullary fixation was applied to 43 long bones in 21 patients due to fracture or deformity. Of these 43 fixations, 14 (33%) were telescopic nails, while 29 (67%) were non-telescopic implants such as Rodsor-Kirschner wires (K-wires). Two types of telescopic implants from the same company and brand were used. While seven (50%) of them were corkscrew-tipped implants (CLT) with a helical locking system in the epiphysis, seven of them (50%) had a locking hole system with K-wire (KLT). The indications for treatment were 25 (86%) deformities and four (14%) fractures in the non-telescopic implant group, compared to nine (64%) deformities and five (36%) fractures in the telescopic implant group (p = 0.124).

Of the non-telescopic implants, nine (31%) were applied to the femur, 16 (55%) to the tibia, two (7%) to the radius, and two (7%) to the ulna, while 14 (100%) of the telescopic implants were applied to the femur (Table 1).

**Table 1:** Distribution of implants concerning bones.

Extremity	Implants		Total
	Telescopic	Non-telescopic	
Femur	14 (100%)	9 (31%)	23
Tibia	0	16 (55%)	16
Radius	0	2 (7%)	2
Ulna	0	2 (7%)	2
Total	14	29	43

While at least one complication was observed in 36 (84%) of 43 operated bones, no complication was encountered in only seven (16%). At least one complication was encountered in nine (64%) of 14 bones with telescopic implants and 27 (93%) of 29 bones with non-telescopic implants (p = 0.028). Considering the major complications requiring surgical treatment, major complications were seen in seven (50%) bones in the telescopic implant group and 27 (93%) bones in the non-telescopic implant group (p = 0.003). The total number of complications and their distribution according to the bones are given in Table 2.

**Table 2:** Distribution of complications by implant and bone.

Extremity	Telescopic implants		Non-telescopic implants		Total
	Major	Minor	Major	Minor	
Femur	9	12	12	9	42 (54%)
Tibia	0	0	27	6	33 (42%)
Radius	0	0	1	0	1
Ulna	0	0	2	0	2
Total	9	12	42	15	78
	21 (27%)		57 (73%)		

**Figure 1:**
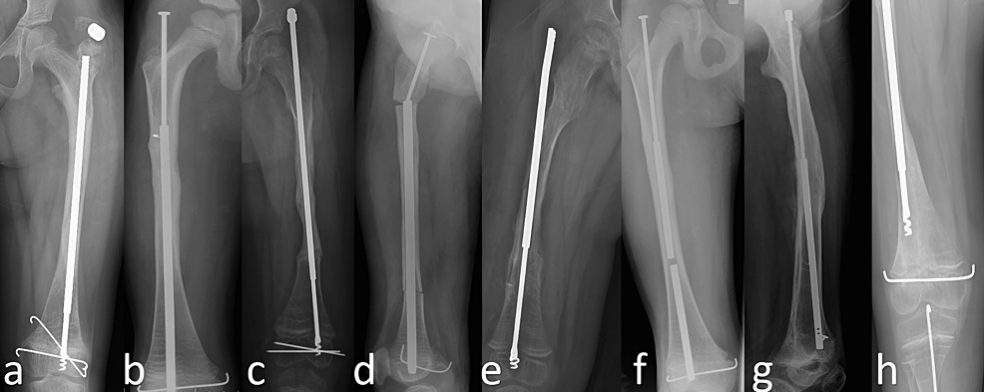
Complications seen in the telescopic implant group. (a) Dislocation of the proximal locking screw; (b-c) migration into the abductor muscles; (d) fracture of the obturator part of the nail; (e) nail extraosseous; (f) fracture of the sleeve part of the nail; (g) limited telescoping of the implant with a locking hole system with K-wire; (h) limited telescoping of the corkscrew-tipped implant

**Figure 2:**
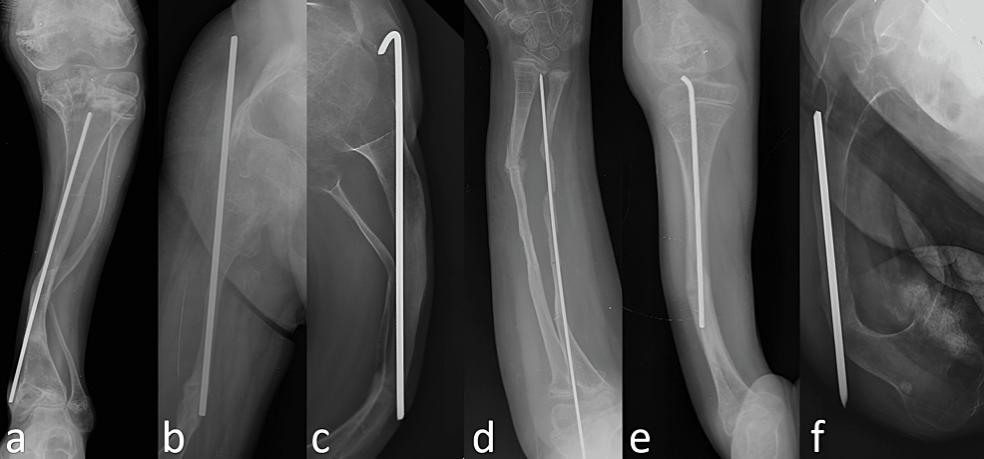
Complications seen in the non-telescopic group. (a) Bone perforation and redeformity; (b) migration into the abductor muscles; (c) migration into the knee joint and redeformity; (d) bone perforation in the forearm; (e,f) bone perforation, redeformity and refracture

Of the 21 complications seen in the telescopic implant group, nine (43%) were major complications (Figure 1). There was no significant difference between the CLT and KLT telescopic implant groups in terms of the total number of operations, the total number of complications, or the number of major complications (p < 0.05). Of the 57 complications seen in the non-telescopic group, 42 (74%) were major complications (Figure 2). While there was no significant difference between the telescopic and non-telescopic groups in terms of the total number of complications (p = 0.342), there was a significant difference between the two groups in terms of the total number of operations and the number of major complications (p = 0.011) (p = 0.002).

Considering the femurs with telescopic and non-telescopic implants due to the fact that all telescopic implants were used in the femur, telescopic implants were used in 14 (61%) of 23 femurs, and non-telescopic implants were used in nine (39%). While the total number of complications in the femur was 42, 21 (50%) of them were in the telescopic implant group and 21 (50%) were in the non-telescopic implant group (p = 0.126). Twenty-one (50%) of the complications observed were determined to be major complications requiring surgical treatment. Of these, 12 (61%) were in the non-telescopic group, and nine (39%) were in the telescopic group (p = 0.041). While there was no significant difference between the femurs in both groups in terms of the total number of complications (p = 176), there was a significant difference between the femurs in the two groups between the total surgeries performed (p = 0.025).

Considering the diversity among the complications, the most common complications were redeformity (29%), bone perforation (26%), and refracture (15%) (Table 3). Migration into the knee joint was seen in six (8%), migration into the abductor muscles five (6%) times, and migration into the ankle joint was not observed in either group. Bone perforation was not observed at all in the telescopic group. However, 20 (35%) of the 57 complications in the non-telescopic group were bone perforations. In the telescopic group, four (28%) of the 14 implants had bending or breakage of the nail fragments, and limited telescoping was observed in three (21%).

**Table 3:** Types of complications according to the implant used.

Complication	Total	Implant	
		Telescopic (percentage of 14 implants)	Non-telescopic (percentage of 29 implants)
Refracture	12	3 (21%)	9 (31%)
Redeformity	23	5 (36%)	18 (62%)
Bone perforation	20	0	20 (69%)
Soft-tissue perforation	2	0	2 (7%)
Migration into the knee joint	6	0	6 (21%)
Migration into the abductor muscles	5	4 (29%)	1 (3%)
Dislocation of the proximal locking screw	1	1 (7%)	0
Migration into the knee joint	1	0	1 (3%)
Fracture of the sleeve part of the nail	1	1 (7%)	0
Bending of the obturator part of the nail	1	1 (7%)	0
Fracture of the obturator part of the nail	2	2 (14%)	0
Limited telescoping	3	3 (21%)	0
Nail extraosseous	1	1 (7%)	0
Total	78	21	57

## Discussion

The main purpose of the development of telescopic implants in the deformity correction and fracture fixation of patients with osteogenesis imperfecta was to accompany the elongated extremity to reduce the complications and repetitive surgeries seen with non-telescopic implants. This study also showed that complications and repetitive surgery requirements are less common with telescopic implants. However, in terms of the total number of complications, the similarity with non-telescopic implants continues. The basis for this is the technical difficulty of implant designs and applications. Similar to our study, Jerosch et al. [10] reported 63.5% and Gamble et al. [7] reported 69% complications with telescopic implants in their series. However, the current literature presents convincing results similar to this study on telescopic implant survival. Cho et al. [12] (88%), Marafioti et al. [13] (77%), and El-Adl et al. [14] shared their series with 92.6% survival rates. Contrary to this, however, Azzam et al. reported that 53% of patients in their series were revised for redeformity and refracture at an average of 52 months [15].

Among the complications, the most common complication seen in both groups is redeformity, which indicates that the osseous pathologies caused by the disease cannot be stopped structurally, regardless of the implant used. In the literature, there are studies stating that this collagen malformation, which cannot be corrected surgically, causes redeformity, refracture, and perforation [16]. In the same series, it was emphasized that approximately 68% of the complications of telescopic implants were of both mechanical and biological origin. Although intramedullary fixation of long bones is preferred over regional plate or temporary percutaneous fixation methods, combining it with additional medical treatments such as bisphosphonates or hormone treatments may prevent the occurrence of these complications. Although current pharmacological drugs slow bone turnover, they do not target the matrix abnormality that is the main cause of the deformity [17-18].

The most common complications of telescopic implants in this series were migration into abductor muscles (29%) and limited telescoping (21%) after redeformity and refracture. The common point between these two complications was the failure of the proximal and distal locking systems. Implant-locking techniques to be made from both ends of a pathological bone have continued to be the problem of all researchers in the historical process. Jerosch et al. reported proximal migration as 15% in their series [10]. Similarly, Lee et al. also reported seven proximal migrations in 50 femoral telescopic implants [19].

In the literature, distal locking differs due to technical difficulties and complications. Both types of distal locking mechanisms in our series had similar locking problems and limited telescoping. In a clinical and biomechanical combined study conducted by Sarıkaya et al., which included the biomechanical comparison of two types of telescopic implants we used in our series, it was emphasized that CLT implants withstand pullout strength better than KLT implants [20]. However, in the clinical part of the study, which included 17 CLT implants, they reported three complications, including limited telescoping, due to the distal locking problem. In the study of Müsiak et al., telescoping arrest was observed in 25 of 58 implants without any mechanical complications, and 16 of them were in the distal male part [21]. In the studies conducted by Lee et al. [19] and Holmes et al. [22], it was emphasized that the most important reason leading to implant migration was the position of the distal end of the rod relative to the epiphysis center, and it was claimed that the deviation in the center reduced the stability of the distal attachment. However, although all implants in our study were central, distal stabilization and limited telescoping problems continued. Shin et al. aimed to solve this problem with the dual locking method and used proximal and distal wire-locking implants in 26 tibias [23]. Although they did not see any sleeve migration in their series, the limited telescoping problem continued.

The bone with the most complications and requiring surgical treatment was the tibia. However, no telescopic implants were used in this series. The most important reason preventing this is the small medullary diameter. Although smaller-scale implant production and its use in the tibia are widespread in the literature, distal and proximal locking problems continue in addition to the fracture or bending problems caused by the thin implant. Cox et al. also reported 21 (77%) complications in 27 tibias in their series [24]. Zionts et al. also stated that complications in the tibia are more common in other regions and that intramedullary implant placement problems are more common in the tibia [25]. Therefore, producing intramedullary telescopic implants with a higher elastic modulus or plate-style extramedullary telescopic implants may be a remedy for this problem.

This study includes some limitations. At the beginning of these, the subgroups of the diagnosis of osteogenesis imperfecta were not examined, the patients could not be standardized in this regard, and genetic research was not carried out. However, it is known that according to the Sillense classification, mild and non-deforming phenotypes are grouped as type 1, perinatal lethal ones are grouped as type I2, severe non-fatal progressive deforming phenotypes are grouped as type 3, and moderately severe phenotypes are grouped as type 4. Type 3 and type 4 patients are the most common in operated patients, and the surgical decision to be made does not differ according to the osteogenesis imperfecta subgroup [26]. Another limitation is that telescopic implants consist of two types of implants, not a single model. Although this reveals the diversity of complications in implants in different systems, a larger series and longer follow-up are needed for a more efficient comparison. The inability to use telescopic implants for the tibia can be considered another limitation. We believe this can be eliminated with a larger series where telescopic implants are applied to the entire lower extremity.

## Conclusions

The use of telescopic implants relatively reduces the complication rate and the need for repetitive surgery in patients with a diagnosis of osteogenesis imperfecta. However, the number of complications is still as high as with non-telescopic implants. In addition, these complications vary according to the implant and technique applied. This study highlights that the search for telescopic implant design and technical application should continue despite all technological developments.
